# Artificial Intelligence for Diabetes Management and Decision Support: Literature Review

**DOI:** 10.2196/10775

**Published:** 2018-05-30

**Authors:** Ivan Contreras, Josep Vehi

**Affiliations:** ^1^ Modeling, Identification and Control Laboratory Institut d'Informatica i Aplicacions Universitat de Girona Girona Spain; ^2^ Centro de Investigación Biomédica en Red de Diabetes y Enfermadades Metabólicas Asociadas Girona Spain

**Keywords:** diabetes management, artificial intelligence, machine learning, mobile computing, blood glucose

## Abstract

**Background:**

Artificial intelligence methods in combination with the latest technologies, including medical devices, mobile computing, and sensor technologies, have the potential to enable the creation and delivery of better management services to deal with chronic diseases. One of the most lethal and prevalent chronic diseases is diabetes mellitus, which is characterized by dysfunction of glucose homeostasis.

**Objective:**

The objective of this paper is to review recent efforts to use artificial intelligence techniques to assist in the management of diabetes, along with the associated challenges.

**Methods:**

A review of the literature was conducted using PubMed and related bibliographic resources. Analyses of the literature from 2010 to 2018 yielded 1849 pertinent articles, of which we selected 141 for detailed review.

**Results:**

We propose a functional taxonomy for diabetes management and artificial intelligence. Additionally, a detailed analysis of each subject category was performed using related key outcomes. This approach revealed that the experiments and studies reviewed yielded encouraging results.

**Conclusions:**

We obtained evidence of an acceleration of research activity aimed at developing artificial intelligence-powered tools for prediction and prevention of complications associated with diabetes. Our results indicate that artificial intelligence methods are being progressively established as suitable for use in clinical daily practice, as well as for the self-management of diabetes. Consequently, these methods provide powerful tools for improving patients’ quality of life.

## Introduction

### Overview

Diabetes mellitus refers collectively to a group of diseases resulting from dysfunction of the glucoregulatory system [1]. Hyperglycemia, the hallmark of diabetes, is the primary consequence of this dysregulation. Chronic hyperglycemia in diabetes is associated with long-term complications involving tissue damage and organ failure, which can decrease life expectancy and even cause death. The International Diabetes Federation estimates that, by 2017, diabetes affected 425 million people worldwide, of whom, 4 million died in the same year. These figures are expected to increase dramatically in the coming decades, placing a rising burden on health care systems [2].

Most diabetes can be categorized into 3 subgroups: type 1 diabetes (T1D), type 2 diabetes (T2D), and gestational diabetes (GDM). Over the long term, T2D patients become resistant to the normal effects of insulin and gradually lose their capacity to produce enough of this hormone. A wide range of therapeutic options are available for patients with T2D. At the early stages of disease, they commonly receive medications that improve insulin secretion or insulin absorption, but eventually they must receive external doses of insulin. On the other hand, T1D patients have severe impairments in insulin production, and must use external insulin exclusively to manage their blood glucose (BG). Treatment of T1D requires consistent doses of insulin through multiple daily injections (MDIs) or continuous subcutaneous insulin infusion (CSII) using a pump. GDM is treated similarly to T2D, but only occurs during pregnancy due to the interaction between insulin and hormones released by the placenta.

In each class of diabetes, timely diagnosis, education of patients in self-management, and continuous medical care are required to prevent acute complications (eg, ketoacidosis) and minimize the risk of long-term complications (eg, nephropathy, retinopathy, diabetic foot, cardiovascular disease, or stroke). In addition to medication, management of diabetes requires adherence to an array of self-care behaviors that are often very burdensome for patients: carefully scheduling meals, counting carbohydrates, exercising, monitoring BG levels, and adjusting endeavors on a daily basis. The effects of nonadherence to recommended treatment are not immediately evident and long-term complications may take years to develop. Accordingly, diabetes therapy is complex, and therapeutic decisions need to take into account diverse medical factors and lifestyle-related activities that must be optimized to improve diabetic patients’ quality of life.

**Figure 1:**
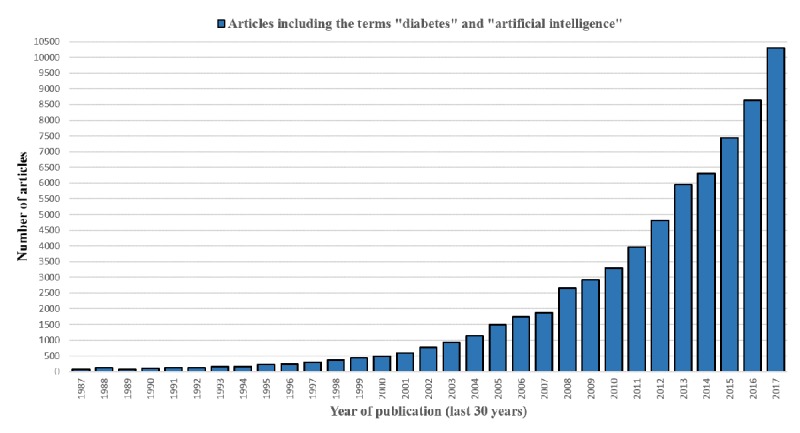
The number of published articles in Google Scholar that include the terms “diabetes” and “artificial intelligence.”.

Artificial intelligence (AI) is a quickly growing field, and its applications to diabetes research are growing even more rapidly as shown in Figure 1, which is a gross estimate of the number of related articles in the Google Scholar database.

In the literature, intelligent algorithms are widely used in data driven methods to support advanced analysis and provide individualized medical aid. There is also evidence that an increasing number of health care companies are applying these techniques [3]. Short-term prospects indicate they are likely to have considerable success in clinical practice. The main reasons for this growth include the explosive increase in the amount of available information, along with the improved performance of intelligent methodologies capable of handling and processing this information, both of which have led to the development of tools and applications which can enhance the effective management of complicated diseases, including diabetes and cancer.

Over the last decade, the entire paradigm of diabetes management has been transformed due to the integration of new technologies such as continuous glucose monitoring (CGM) devices and the development of the artificial pancreas (AP), along with the exploitation of data acquired by applying these novel tools. AI is attracting increased attention in this field because the amount of data acquired electronically from patients suffering from diabetes has grown exponentially. By means of complex and refined methods, AI has been shown to provide useful management tools to deal with these incremental repositories of data. Thus, AI has played a key role in the recognition of these systems as routine therapeutic aids for patients with diabetes.

The literature offers ample evidence of the use of artificial intelligence methods in the field of diabetes, such as in general surveys [4,5] or in particular domains, for example early diagnosis [6]. In this manuscript, we describe the latest efforts and advances in the application of AI methodologies to diabetes management and decision support. Background information on AI methods is provided in the remainder of the Introduction section.

We then provide a detailed description of the methodological approach used in this review, report the results of a literature analysis, discuss studies on various sub-topics, and conclude with a brief summary and a discussion of future challenges.

### Artificial Intelligence Techniques

**Figure 2:**
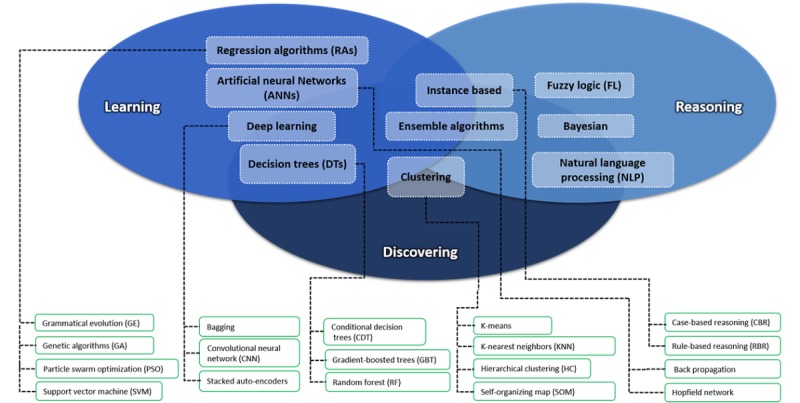
A taxonomy of some of the best known artificial intelligence methods.

Defining the concept of AI, computational intelligence, or machine intelligence is not a trivial undertaking. In this paper, we refer to AI as a branch of computer science that aims to create systems or methods that analyze information and allow the handling of complexity in a wide range of applications (in this case, diabetes management). Although the application of AI algorithms involves highly technical and specialized knowledge, this has not prevented AI from becoming an essential part of the technology industry and making contributions to major advances within the field. This section will provide a short overview of several well-known computational intelligence paradigms. For a more in-depth discussion of various intelligent algorithms, theoretical results, and applications, the reader is referred to the following book by Nilsson [7]. In this study, we categorized methodologies with respect to the objective sought: to explore and discover information, to learn using information, or to extract conclusions from information (see Figure 2).

#### Learning from Knowledge

**Figure 3:**
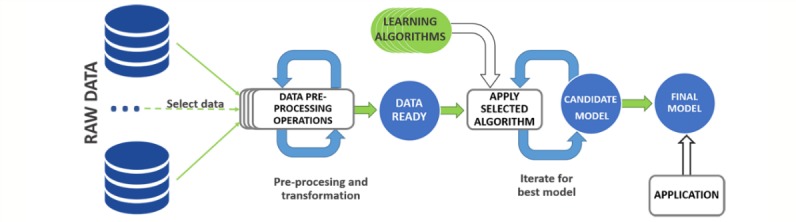
A general diagram of the learning algorithm process.

Acquisition of knowledge is a key requirement of solutions intended to exhibit intelligent behavior. Because learning is an effective way to introduce such knowledge, most AI studies to date have employed learning techniques (see Figure 3). The primary aim of learning from knowledge is to allow computers to learn automatically without human intervention or assistance. This process could involve any method that includes some inductive component, ranging from a simple Kalman filter to a complex convolutional neural network. No method is inherently better than any other; each is more or less well-suited to different scenarios, for example, a softer learning curve, faster execution, or more flexible solutions. Furthermore, the performances of various methods are closely related to the quality and quantity of data: when more information is gathered, and less noise is present in the data, better solutions can be obtained. The most important families of techniques are artificial neural networks (ANNs), support vector machines (SVMs), random forest (RF), evolutionary algorithms (EAs), deep learning, Naïve Bayes, decision trees, and regression algorithms.

#### Exploration and Discovery of Knowledge

The discovery of knowledge revolves around the exploration and creation of algorithms for retrieving potential information from databases, commonly referred to as knowledge discovery in databases (KDD). The primary objective of KDD is identification of valid, potentially useful, and understandable information. KDD involves evaluation and interpretation of patterns and models for making decisions about what does and does not constitute knowledge, that is, distinguishing between data that are useful and those that are (in the context of interest) useless. Therefore, KDD requires broad and deep knowledge about the area of study.

**Figure 4:**
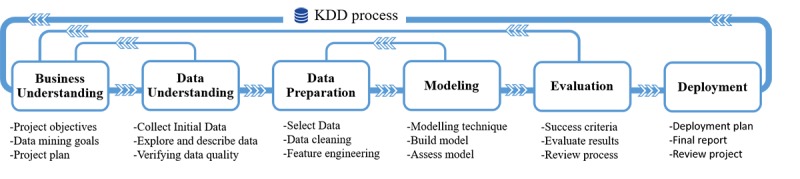
General CRISP-DM model for the knowledge discoveryin databases (KDD) process.

The overall KDD process may be characterized into 6 steps in the cross industry standard process for data mining (CRISP-DM) model (Figure 4) [8]: business understanding, data understanding, data preparation, data modeling, evaluation of the model, and deployment. The application of data mining modelling is the most technical stage of the process. Techniques for data mining have taken much of their inspiration from learning algorithms and statistics, although the two types of approaches have different objectives. The most important data mining tasks involve the detection of anomalies, identification of dependencies between variables, regression, clustering, and classification. Some examples of representative techniques for this process are k-means, the k-nearest neighbor algorithm, and hierarchical clustering (HC).

#### Reasoning from Knowledge

In this discourse, the idea of reasoning from knowledge denotes the creation of precise and effective ways to generate inferences in more precise and robust ways. Thus, reasoning from knowledge involves the use of logical techniques such as deduction and induction to generate conclusions from the available knowledge. The primary objective of systems that implement reasoning mechanisms is to perform tasks at a human-expert level in a narrow, specialized manner within the domain of interest. Such systems commonly apply heuristics to guide reasoning and reduce the search space of possible solutions.

**Figure 5:**
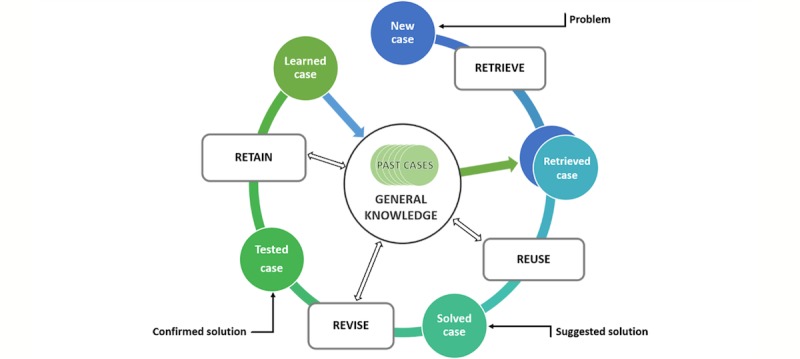
The case-based reasoning circle.

These systems are based on 3 main components. First, a knowledge acquisition system is used to gather and collect inferences that can be used for further development. In this context, such a system is used to extract new rules and gather information. Second, a knowledge base, characterized by rules and information, is used for problem solving. Important aspects here include relations, conditions, recommendations, directives, and strategies. Finally, the inference engine links the knowledge base with the gathered information. Overall, this process facilitates reasoning, whereby the system becomes able to facilitate the realization of the anticipated solution. It is possible for a system structured on this basis to transfer expert knowledge directly to the knowledge base. This, in turn, helps to build new solutions based on previous cases, or to deal with ambiguous concepts and uncertainty. Representatives of these tasks include rule-based reasoning, case-based reasoning (illustrated in Figure 5), and fuzzy logic.

## Methods

A review of the literature was conducted using the PubMed database. The selection of this bibliographic system as the primary data source was motivated by the sharp increase in the number of articles in the database and the strong link between these articles and the health care sector. PubMed has been validated as a reliable tool for retrieving information on medical research and clinical applications. Only English-language documents published between 2010 and 2018 were considered. The search terms listed in Textbox 1 were used to identify terms in the abstracts, titles, and keywords of the documents.

The search terms were explored and combined, yielding 1841 “hits.” The terms “diabetes,” “management,” “artificial pancreas,” and “blood glucose” were combined with the remaining terms using a conjunctive operator, and these terms were used as keywords to create individual datasets comprising all references to the following phrases: “artificial intelligence” (186), “computational intelligence” (179), “machine learning” (88), “data mining” (111), “deep learning” (3), “k-means” (9), “fuzzy logic” (24), “heuristic” (10), “clustering analysis” (281), “Bayes” (19), “decision tree” (67), “random forest” (21), “particle swarm optimization” (7), “pattern recognition” (31), “genetic algorithm” (43), “supervised algorithm” (14), “unsupervised algorithm” (9), “knowledge-based” (14), “case-based reasoning” (11), “decision support system” (71), “self-organizing map” (4), “evolutionary computation” (2), “neural network” (72), “natural language processing” (34), “reinforcement learning” (6), clustering (510), and “support vector machine” (23). Each of these datasets was then combined to build an objective dataset of articles. A comprehensive review was performed of all references cited in the datasets. Finally, the bibliography of the reviewed articles was thoroughly explored to find titles with relevance to the main focus of this study.

**Figure 6:**
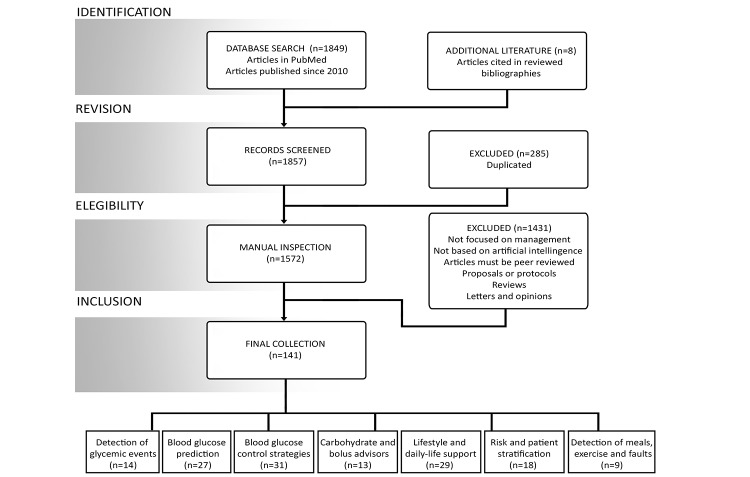
Summary of the review process and classification of articles into a set of subdomains.

The complete method is summarized in Figure 6. The resultant final collection of articles was divided into various categories, designed to assist in the grouping of studies according to their shared and specific characteristics. Over the course of the systematic review, the subcategories were fused and fixed to accommodate the merging of information.

**The terms used in the search queries.**

Artificial intelligence; Artificial neural network; Artificial pancreas; Blood glucose; Case-based reasoning; Cluster analysis; Clustering; Computational intelligence; Data mining; Decision support systems; Decision tree; Deep learning; Diabetes; Evolutionary computation; Fuzzy logic; Genetic algorithm; Heuristic; K-means; Knowledge-based; Machine learning; Management; Natural language processing; Naïve Bayes; Particle swarm; Pattern recognition; Random forest; Reinforcement learning; Self-organizing map; Supervised learning; Support vector machine; Unsupervised learning

## Results

### Main Findings

Ultimately, 141 papers were included in the review. The potential of AI to enable diabetes solutions has been investigated in the context of multiple critical management issues. In this section, we use the following proposed diabetes management categories to summarize the latest contributions and the results described in the reviewed articles:

- Blood glucose control strategies
- Blood glucose prediction
- Detection of adverse glycemic events
- Insulin bolus calculators and advisory systems
- Risk and patient personalization
- Detection of meals, exercise and faults
- Lifestyle and daily-life support in diabetes management

**Figure 7:**
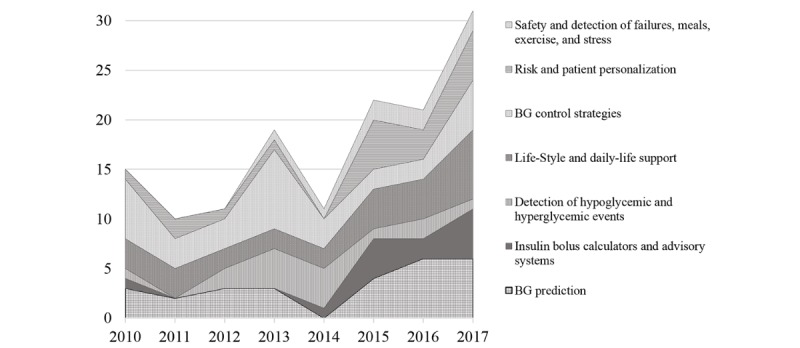
Number of articles reviewed according to subdomain and year of publication (BG: blood glucose).

As seen in Figure 7, the majority of the papers were published in the last 3 years, reflecting a clear acceleration in the application of AI techniques to diabetes management. In the following, contributions to each of the subdomains are detailed and discussed.

### Blood Glucose Control Strategies

Development of the AP has been intensively pursued over the past decade. An AP consists of an automated system that mimics islet physiology, including a glucose sensor, a closed-loop control algorithm, and an insulin infusion device. The ultimate goal of an AP system is to improve overall diabetes management and reduce the frequency of life-threatening events associated with T1D. The algorithms used by the AP to calculate insulin dosage have been intensively investigated, either using data from diabetic patients or computer simulated patients, commonly named virtual patients (VP). The major candidate algorithms are derived from traditional control engineering theory; however, AI has become more established over the past few years and could ultimately provide better candidates to meet the challenges of an AP [9].

Although AI and control engineering have converged to some extent as the two fields incrementally exchange methods, here we will focus on studies dealing with closed-loop algorithms based on AI techniques. We direct interested readers to a recent comprehensive review on AP systems [10].

Three main AI methodologies have been established as control techniques in recent years: FL, ANNs, and reinforcement learning (RL). Most alternatives to control engineering algorithms are based on FL. Controllers apply FL theory to imitate the lines of reasoning of diabetes caregivers. Thus, the primary benefit of FL over classic control engineering is the ability to deal with nonlinearities and uncertainties. However fuzzy logic systems have not yet been proven to clearly outperform well-tuned classical approaches.

MD-Logic [11,12] was developed by authors who sought to individualize glycemic control using a fuzzy controller. Two feasibility studies were conducted in cohorts of 7 T1D patients to introduce the methodology and test the viability of the controller. Subsequently, a randomized crossover trial was conducted in 12 T1D patients [13]. The results suggested that the fuzzy method could improve nocturnal BG control without increasing the risk of hypoglycemia. Following the success of these feasibility studies, the authors performed a randomized crossover study of 56 young patients over 3 days [14]. The results confirmed a reduced rate of nocturnal hypoglycemia and superior glycemic control in comparison with insulin pump treatment. In a home-based randomized trial of 15 T1D patients [15], the authors compared the fuzzy AP and sensor-augmented pump over 4 nights; the results confirmed the feasibility, safety, and efficiency of their approach in a home setting. Later, an extended study of 24 T1D patients during 6 weeks of nocturnal control demonstrated the safety and effectiveness of long-term use of a FL-based controller. In a recent clinical trial evaluating remote patient monitoring of the FL controller, the AP was tested in 75 T1D patients for 4 consecutive nights. The results demonstrated safe and efficient glycemic control. Further studies will evaluate the MD-Logic controller implemented in MiniMEd 690G [16].

Other research groups have also investigated the application of FL to BG control. For example, Mauseth et al [17] reported a FL controller designed to personalize glycemic control. They tested it in 30 virtual patients on the UVA/Padova T1D simulator. Next, to demonstrate the feasibility of their approach, they conducted a pilot study in 12 T1D patients [18]. In a later study, they proposed stressing a fuzzy controller with high-fat meals and exercise and tested this approach in a trial with 10 T1D patients [19]. The results revealed deficits in their previous approach and ultimately led to improvements in the FL controller.

Other FL approaches have been tested using virtual patients or simulations [20-24]. For example, Miller et al reported a fuzzy controller combined with a learning algorithm that extracted initial patient profiles using open-loop data [23]. Another example was provided by Dinani et al [24], who suggested combining fuzzy and sliding-mode controllers, with the goal of using feedback to govern the insulin delivery rate more aggressively.

Another AI method that has been increasingly adopted in the area of control algorithms is the so-called reinforcement algorithm [25]. Daskalaki et al presented an adaptive, patient-specific BG control strategy based on the Actor-Critic learning approach and tested their approach on 28 virtual T1D patients (adults, adolescents, and children) [26].

Another AP study based on RL was proposed by Daskalaki et al, who proposed using an Actor-Critic algorithm to optimize insulin infusion for personalized glucose regulation, and evaluated the system using virtual patients [27]. The results revealed that their novel tuning method decreased the risk of severe hypoglycemia, especially in patients with low insulin sensitivity. Other AI-related techniques used to support the development of the AP included modeling of glucose metabolism with an SVM [28].

Over this period, several groups proposed complementary AI algorithms to support AI controllers. Fereydouneyan et al proposed assisting the controller with a genetic algorithm (GA) that optimizes the values for two inputs and one output membership function [22], with the goal of preventing fluctuations caused by derivatives in fuzzy design. Another GA was used in the work of Catalogna et al to support an ANN controller [29]. In that case, the proposed GA optimizes network topology and learning features, instead of using the trial-and-error approach commonly adopted in ANN topology determination. In line with previous studies, Khooban et al proposed a controller assisted by particle swarm optimization that optimizes the parameters of the glucose-insulin model [21]. Following the trend of combining methods to control BG with metaheuristics, Yadav et al proposed the use of a Cuckoo search algorithm to optimize the gain of the controller [20]. Optimization of AI techniques, aside from their use in conjunction with AI controllers, have been used in other studies to determine controller parameters. For example, Tang et al used GA to mine information from patients’ medical histories to generate multiple customized models [30], and Greenwood et al proposed the use of particle swarm optimization to adjust the function cost of an economic predictive control model [31].

Complementary to advances in control algorithms, efforts have been made to improve models that attempt to capture glucose-insulin dynamics. Since the publication of the seminal paper in this field [32], interest has increased in applying neural networks to identification and control of nonlinear systems. Zarkogianni et al [33,34] developed a recurrent neural network trained with a real-time recurrent learning algorithm that models the BG kinetics of T1D patients and predicts BG levels using information about meal intake, BG measurements, and infused insulin. González-Olvera et al investigated the use of a fuzzy neural network in an attempt to combine the best properties of ANNs and fuzzy systems [35]. Specifically, they implemented a learning system that combines input signals, infused insulin, and BG measurements with a membership initialization based on the fuzzy c-means algorithm. The following year, Alanis et al performed a more rigorous study using a recurrent neural network trained to model BG in T1D patients [36]. The approach they implemented considered glucose absorption via carbohydrates consumption and insulin infusion as inputs. These 3 neural network studies proposed a model of insulin dynamics as the first step in the design of a control scheme, and then validated the model using T1D patient data. Their results demonstrated that they were indeed able to capture BG dynamics. More recently, 2 studies presented a complete control scheme using neural networks [29,37]. Both studies sought to determine whether this technique could predict and control BG excursions in T1D using patient-specific models. The first approach was tested using the hyperinsulinemic-euglycemic clamp technique in 34 rats, and the second was tested using *in silico* data including BG measurements, administered insulin, exercise information, and ingested food. The results of both approaches demonstrated that ANNs are accurate methods for regulation of glucose levels.

### Blood Glucose Prediction

The ability to anticipate BG excursions could provide early warnings regarding ineffective or poor treatments. Thus, information collected from new technologies for diabetes management, such as the CGM devices, could lead to real-time predictions of future glucose levels. Prediction of BG levels is challenging due to the number of physiological factors involved, such as delays associated with absorption of food and insulin, and the lag associated to measurements in the interstitial tissue. Errors of the CGM also increase the difficulty of predicting BG values (approximately 9% of the mean absolute relative difference for the best sensors [38]).

The results of this section are presented in Table 1, which captures the critical information from all studies in which AI methods were used to predict BG values. The table was designed to provide quick access to information about current technologies being tested. We outline the features of each study using key information, including prediction horizon (PH) in minutes, objective population criteria, number of participants in the cohort, mean number of monitored days per patient, mean number of monitored hours per day, existence of monitoring during the overnight period, type of monitoring technology, and information about physical activity.

**Table 1:** Summary of reviewed studies addressing blood glucose prediction: prediction horizon in minutes, objective population criteria, number of participants in the cohort, mean number of monitored days per patient, mean number of monitored hours per day, type of monitoring technology, existence of monitoring during the overnight period (O) and inclusion of exercise or physical activity information (E).

Prediction horizon (min)	Population	Cohort	Days	Time	O	E	Method	Ref	Year
15, 30, 45	T1D^a^	15	28	10 h	✓		ANN^b^	[39]	2010
30	T1D	12	10	24 h	✓		ANN	[33]	2010
75	Critical Care	1	16	15 h	✓		ANN	[40]	2010
75	T1D	27	5	24 h	✓		ANN	[41]	2011
30	T1D	5VP^c^, 1	7	24 h	✓	✓	ANN	[42]	2011
30, 45	T1D	30VP	8	24 h	✓		ANN	[43]	2012
15, 30, 60, 120	T1D	27	13	24 h	✓	✓	RF^d^	[44]	2012	
30	T1D	20VP, 9	11, 7	24 h	✓		ANN	[45]	2012
30	T1D	10	3	24 h	✓		SVM^e^, RA^f^, ANN	[46]	2013
15, 30, 45	T1D	23	6.1	24 h	✓		RA, ANN	[47]	2013
15, 30, 60, 120	T1D	27	13	24 h	✓	✓	SVR	[48]	2013
30	T1D	20	3	24 h	✓		ANN	[49]	2015
15, 30, 45, 60	T1D	6	11	24 h	✓	✓	ANN	[50]	2015
30, 60, 120	T1D	10	6	24 h	✓	✓	ANN	[51]	2015
30	T1D	15	13	24 h	✓	✓	ANN	[52]	2015
30	T1D	5VP, 1	30	24 h	✓		SVR	[53]	2016
—	T2D^g^	346	1	—			ANN	[54]	2016
5, 15, 30, 45, 60	T1D	15	13	24 h	✓	✓	Kernel	[55]	2016
60	T1D	5	90	24 h			EA^h^	[56]	2016
1440	T1D, T2D	8	3	24 h		✓	DT^i^	[57]	2016
30	T1D	3	10	24 h	✓		EA	[58]	2016
30,60	T1D	17	6	24 h	✓		RA	[59]	2017
60, 120, 150, 180	T1D	20VP	14	24 h	✓		EA	[60]	2017
0	T2D	3	23	—			NB^j^	[61]	2017
30, 60, 90, 120	T1D	10	10	24 h	✓		KNN^k^, RF, EA	[62]	2017
120	T1D	100VP	14	24 h	✓		EA	[63]	2017
30, 60, 90	T1D & T2D	106	<7	24 h	✓		RA and ANN	[64]	2017

^a^T1D: type 1 diabetes.

^b^ANN: artificial neural network.

^c^VP: virtual patient.

^d^RF: random forest.

^e^SVM: Support Vector Machine

^f^RA: regression algorithm.

^g^T2D: type 2 diabetes.

^h^EA: evolutionary algorithm.

^i^DT: decision tree.

^j^NB: Naïve Bayes.

^k^KNN: k-nearest neighbor.

Finally, we highlight the main AI methods applied in the studies, the bibliographic reference, and the year of publication. AI for BG prediction has been addressed in as many as 13 parallel lines of research. Most of these studies focused on T1D because of the inherent utility of AI in this condition and the availability of high-frequency data collected from patients using a CGM device. The results of our review reveal the range of PHs explored, from 5 to 180 minutes. Short-term predictions were the most frequently explored: 38 out of 49 studies (76%) used PHs below 60 minutes. ANN approaches were the most widely applied methodology, but other machine learning methodologies such as RF, SVM, or RAs are being adopted with increasing frequency.

### Detection of Adverse Glycemic Events

As with BG prediction, glycemic episode detection encompasses a set of tools that deal with the complexity of effective BG control. However, in this section we will not address glucose values, but instead focus on the appearance of hyperglycemic or hypoglycemic events. These tools enable us to detect the occurrence of glycemic episodes and give us the opportunity to respond promptly to their effects. In contrast to the previous section, most of the reviewed studies on this topic focus on detecting hyperglycemia or hypoglycemia in situations when it is not possible to effectively monitor BG. Therefore, most of these studies deal with real-time approaches rather than predictions of future events. We summarize the studies dealing with detection of BG excursions in Table 2. Each scenario is represented by the following features: prediction horizon in minutes, objective population criteria, number of participants in the cohort, mean number of monitored days per patient, mean number of monitored hours per day, type of monitoring technology, existence of monitoring during the overnight period, and inclusion of exercise or physical activity information. Finally, we highlight the main AI methods applied in each study, the bibliographic reference, and the year of publication. The results revealed that nine of the 14 studies (64%) reported real-time detection systems, and 10 of these studies (93%) were specifically focused on T1D. Table 2 shows that over six of the approaches that exclusively addressed T1D (60%) gathered data from CGM sensors, whereas the remainder used an electroencephalogram (EEG) or self-monitoring blood glucose (SMBG) measurements. Studies focusing on T1D were performed with fewer than 15 patients, whereas studies of T2D included larger cohorts. Sensitivity and specificity were the most common outcomes used to assess the quality of approaches to glycemic detection. Although this section contains fewer papers than the one on BG prediction, we identified more than 10 research groups contributing to this topic. In particular, researchers at the Centre for Health Technologies (Faculty of Engineering and Information Technology, Sydney, Australia) have published five studies on this topic over the last 7 years.

### Insulin Bolus Calculators and Advisory Systems

The most common insulin therapies for diabetics, continuous subcutaneous insulin infusion (CSII) and multiple daily insulin injections (MDI), operate according to similar principles [79]. Both utilize basal insulin (injection of long-acting basal insulin and infusion at a constant basal rate, respectively) and bolus insulin (injection of quick-acting bolus insulin and meal boluses, respectively) to cover meals or snacks. The calculation of correct insulin doses and the estimation of the amount of carbohydrates is a regular task in the daily life of many insulin-dependent patients. Bolus advisors are based on previous insulin doses, BG measurements, planned carbohydrate estimates, and other patient-specific parameters, including insulin-to-carbohydrate ratio and insulin sensitivity. Manually calculating bolus doses and counting carbohydrates can be complex and challenging because individuals must consider multiple parameters to achieve satisfactory glucose control, and miscalculation of these values may result in persistent glycemic episodes.

To support carbohydrate estimation and determination of insulin doses by patients, tools for providing bolus advice and carbohydrate estimates are increasingly being adopted. These tools seek to increase the accuracy of mealtime and correction boluses. AI has been used to provide sets of tools to improve the accuracy of carbohydrate estimates and to calculate the optimal insulin bolus for the ingested meal

We identified several studies that applied AI to systems aimed at supporting patient decisions by issuing advice regarding meals, exercise, or medication. Research groups at the Imperial College London performed an extensive study of an insulin bolus calculator based on case-based reasoning methodology [80-84]. Their approach, which manages various dynamically optimized diabetes scenarios, was proven in a clinical trial (NCT02053051) to be a safe decision support tool. Additionally, this approach was demonstrated to improve glycemic control in diabetes management when it was combined with an AP system [84]. A similar approach was presented recently by another group [85], which also proposed an insulin bolus calculator based on case-based reasoning but, in contrast to other bolus calculators, it used a novel temporal retrieval algorithm. The Center for Biomedical Engineering Research at the University of Bern performed several important and extensive studies [86-90] investigating the GoCARB system, which provides dietary advice to diabetic patients based on automatic carbohydrate counting. Their approach is based on the use of computer vision techniques, such as feature extraction and SVM, and pilot studies show it to be an excellent assistive tool. We have also found several studies that validated their approach using the UVA/Padova patient simulator. Srinivasan et al proposed the use of a set of insulin delivery profiles optimized by a PSO to find the optimal open- and closed-loop profiles for various meal compositions [91]. More recently, another study [92] presented an approach based on ANN to optimize bolus calculation by patients using CGM. The results revealed that it was better at reducing the blood glucose risk index value than other approaches. Finally, Lee et al proposed an advisory treatment system that provides insulin, meal, and exercise recommendations [93]. Their study, which compared rule-based reasoning and k-nearest neighbor algorithms, concluded that the k-nearest neighbor algorithm was best suited to this approach.

### Risk and Patient Stratification

Most commercially available tools and protocols for managing diabetes are based on general models of the diabetic population or involve subsets of patients defined by simple clusterization features and easily identifiable characteristics. However, the daily lives of diabetic patients are determined by a wide range of management scenarios that are not represented in these general models. Insulin-dependent patients must manage a highly complex process to maintain suitable levels of BG.

**Table 2:** Summary of reviewed studies addressing detection of adverse glycemic events: prediction horizon (PH) in minutes, objective population criteria, number of participants in the cohort, mean number of monitored days per patient, mean number of monitored hours per day, type of monitoring technology, existence of monitoring during the overnight period (O), and inclusion of exercise or physical activity information (E),

PH (min)	Population	Cohort	Days	Time	Measurements	O	E	Method	Ref^a^	Year
0 min	T1D^b^	6	1 day	10 h	EEG^c^	✓		ANN^d^	[65]	2010
0 min	T1D	30	80-247 days	—	SMBG^e^		✓	RF^f^, SVM^g^	[66]	2012
0 min	T1D	15	1 day	10 h	CGM^h^	✓		ANN, PSO^i^	[67]	2012
0 min	T1D	10	30 days	4 h	CGM			SVM	[68]	2013
30, 60 min	T1D	15	12.5 days	24 h	CGM	✓	✓	SVM	[69]	2013
0 min	T1D	10	4.5 days	6 h	CGM			SVM	[70]	2013	
30 min	T1D	10	17.3 days	24 h	CGM	✓		DT^j^	[71]	2013
24 h	T2D^k^	163	—^l^	—^l^	SMBG			RF	[72]	2015
0 min	T1D	15	1 day	4 h	CGM			ANN	[73]	2014
0 min	T1D	10	—^m^	—^m^	SMBG, ECG	✓		ANN	[74]	2014
2, 7, 30, 61-90 days	T1D, T2D	201, 323	—^n^	—^n^	SMBG			Pattern recognition	[75]	2014
0 min	T1D	15	1 day	10 h	ECG	✓	✓	ANN	[76]	2016
Past events	T2D	119695	>12 days	—	EHR^o^			NLP^p^	[77]	2016
0 min	T1D, T2D	500	1 day	2 h	SMBG			DT, ANN	[78]	2017

^a^Ref: reference.

^b^T1D: type 1 diabetes.

^c^EEG: electroencephalogram.

^d^ANN: artificial neural network.

^e^SMBG: self-monitoring blood glucose.

^f^RF: random forest.

^g^SVM: support vector machine.

^h^CGM: continuous glucose monitoring.

^i^PSO: particle swarm optimization.

^j^DT: decision tree.

^k^T2D: type 2 diabetes.

^l^344 data points.

^m^18 data points.

^n^787 data points.

^o^EHR: electronic health record.

^p^NLP: natural language processing.

Treatment of diabetes is governed by diverse factors, implying high intra- and interpatient variability [94]. Exercise, nutrition disturbances, age, and cardiovascular complications are just some of the long list of factors that can dramatically impact quality of life and undermine medication adherence even when patients follow their treatment regimen strictly. Such patient variability severely limits the use of general models, which cannot capture the specific physiological behaviors of individuals. Thus, an important step toward better risk detection and intervention is personalization of the system. Over the past decade, major research efforts have been devoted to developing management tools capable of stratifying patients in different segments of the population. Risk assessment and patient stratification methods are important to improving the management of diabetes, and therefore the overall health outcomes of diabetic patients, and consequently have attracted a greater share of attention from the medical community.

This category gathers all reviewed papers that systematically identified individual patients and their risk factors to manage and coordinate their care based on specific conditions and on evidence-based guidelines. Table 3 outlines the type of stratification together with the specific challenge. Main characteristics, such as number of years, cohort, and objective population, are also included. Finally, the table defines the AI methodology applied, bibliographic reference, and year of publication.

**Table 3:** Summary of studies addressing risk and patient stratification.

Stratification	Challenge	Period	Cohort	Population	Methods	Year	Ref^a^
Complications	Group risks of retinopathy	5 years	55	T1D^b^	DT^c^, ANN^d^	2010	[95]
Disease complexity	Group combinations of comorbid conditions	2 years	15480	Chronic diseases	Hierarchical clustering	2011	[96]
Disease complexity	Group management profiles	3 months	239	T1D	Hierarchical clustering	2011	[97]
Disease complexity	Group management profiles	3.5 months	70	T2D^e^	K-means	2012	[98]
Complications	Group biomechanical foot profiles	6 months	97	T1D, T2D	K-means	2013	[99]
Disease complexity	Group by drug purchases	7 years	953	T2D	Knowledge discovery	2015	[100]
Complications	Group risks of renal disease	3 years	109	T2D	K-means	2015	[101]
Complications	Group risks of complications	10 years	1441	T1D	Learning models	2015	[102]
Complications	Group risks of complications	5 years	84	T1D, T2D	RA^f^ and ANN	2015	[103]
Complications	Group risks of retinopathy	<1 year	345	T2D	SVM^g^, RF^h^, DT, NB^i^	2015	[104]
Disease complexity	Groups of blood glucose profiles	4 months	10	T1D	Hierarchical clustering	2016	[105]
Complications	Group personal networks types	5 years	1862	T2D	RA, K-means;	2016	[106]
Disease progression	Group risks of T2D progression	5 years	24331	T2D	NB	2016	[107]
Complications	Group risks of retinopathy	2 years	323378	T2D	RF	2017	[108]
Disease complexity	Group blood glucose profiles	2 years	27005	T2D	RF	2017	[109]
Disease complexity	Groups of HbA1c profiles	5 years	684	T2D	RAs, KNN	2017	[110]
Weight intervention	Group of BMI profiles	31 years	2540	T2D	RA	2017	[111]
Complications	Group by retinopathy, neuropathy, or nephropathy	3,5,7 years	943	T2D	RF, RA	2018	[112]

^a^ref: reference.

^b^T1D: type 1 diabetes.

^c^DT: decision tree.

^d^ANN: artificial neural network.

^e^T2D: type 2 diabetes.

^f^RA: regression algorithm.

^g^SVM: support vector machine.

^h^RF: random forest.

^i^NB: Naïve Bayes.

### Detection of Meals, Exercise, and Faults

Because people with both types of diabetes need support to successfully manage their disease, solutions with higher accuracy that require less user interaction are associated with higher-quality diabetes treatments. Tools or algorithms capable of early detection of critical events affecting glycemic control, such as exercise, a meal, or an infusion set failure, are critical for systematic automation of both closed-loop and open-loop systems. Insulin-dependent patients monitoring their glucose with CGM devices use BG measurements to calculate insulin infusion rates. Consequently, failure of these devices can lead to episodes of hyperglycemia or hypoglycemia. Leal et al proposed an approach using SVM to detect correct and incorrect measurements in real-time CGM [113]. They tested their system on 23 critically ill patients and obtained promising results in patients with sepsis or septic shock. The same objective was pursued in the work performed by Turksoy et al [114], who used a k-nearest neighbor algorithm for the diagnosis of faults and the data from 51 patients to validate the performance of their approach. For the detection of inaccurate measurements by glucose meters, another study [115] developed an SVM algorithm to minimize the effect of hematocrit on glucose measurement and tested their method on 400 BG samples.

Physical activity offers multiple benefits for diabetic patients, but also complicates the management of diabetes, especially in T1D patients. Some of the factors affecting BG dynamics during exercise include the intensity, duration, and type of exercise, insulin on board, and the carbohydrate absorption rate. Tools and systems focused on automated detection of exercise could improve the accuracy of treatments. Turksoy et al also proposed the use of a k-nearest neighbor classification algorithm to automatically detect exercise type and intensity in an AP system [116]. They tested their approach in 5 T1D patients and reported a sensitivity of 98.37%. Similarly, Jacobs et al proposed a regression model to automatically detect physical exercise in patients carrying an accelerometer and a heart rate sensor [117]. The system was assessed in 13 T1D patients, yielding a sensitivity of 97.2% and a specificity of 99.5%.

Meal detection is important in AP systems that do not permit manual meal announcements and as a safety system for patients who may forget to enter meal information manually. Turksoy et al have also investigated the development of a meal detection system based on analysis of CGM signals using an unscented Kalman filter and a fuzzy system to estimate the carbohydrates content [118-120]. Their approach was validated *in silico* with 30 T1D patients using the UVA/Padova simulator, which revealed a sensitivity of 91.3% and an error of 23.1% in carbohydrate estimation; and *in vivo* using data from 11 T1D patients, which revealed a sensitivity of 93.5% for meals and 68.0% for snacks.

Insulin pump failure may result in prolonged hyperglycemia or diabetic ketoacidosis. Early detection of failures could minimize the associated risk. Cescon et al proposed the use of a time-varying autoregressive model to develop a patient alert system [121]. Validation with data from 9 T1D patients during 18 weeks of infusion set wear revealed that the system had 50% sensitivity and 66% specificity.

### Lifestyle and Daily-Life Support in Diabetes Management

Lifestyle management is a fundamental aspect of diabetes care. Sedentary living, stress, nonadherence to medication, lack of regular medical examinations, and bad habits can lead to discontinuation of treatment for patients with diabetes. From the time of diagnosis, patients are required to optimize their lifestyles to manage complications and other comorbid conditions, with the overall goal of enhancing their own care. Current technologies and data warehouses enable solutions that model data and make quality decisions based upon them. Decision support systems (DSSs) consist of tools focused on helping patients or doctors to manage diabetes therapies. These systems usually have monitoring features that facilitate systematic recording of information about diet, physical activity, medication, glucose measurements, etc and combine it with tools to support both patients and clinicians, with the overall goal of enhancing therapeutic outcomes.

Multiple studies aimed at developing DSSs to manage diabetes have been proposed since 2010. One of the most productive approaches is the METABO project [122-127]. This project involves monitoring and advanced features including tools to prevent future excursions, dynamically optimize care pathways, extract patterns via knowledge discovery, and guide weight loss programs. The authors conducted several pilot trials, including usability tests in 36 T1D patients. The MOSAIC project [100,110,112,128], another important project, is focused on the development of a DSS for T2D management, with a special focus on the risk assessment of related complications using data mining methods. Another daily-life support system has advanced tools, such as a recommender system that employs case-based reasoning and an integrated BG prediction tool based on evolutionary computation [129].

Recently, Everett et al presented a DSS using machine learning to promote adherence to physical activity and weight reduction [130]. Authors validated the system with 55 patients with prediabetes. Previously, Yom-Tov et al proposed a DSS based on a RL algorithm that automatically sends messages to patients who are following a personalized plan for physical exercise [131]. The approach was validated in 27 sedentary T2D patients. Daily-life support systems using AI tools for GDM were also investigated. A weight management proposal was presented in the MediClass system [132]. The system, which is based on the application of a natural language processing (NLP) algorithm, was validated during the postpartum visits of 600 GDM patients. Rigla et al also investigated tools for GDM patients [133]. They proposed a mobile app based on an AI-augmented telemedicine DSS as a tool for helping GDM patients. Later, they presented a platform to remotely evaluate patients using a classifier based on a clustering algorithm and a decision tree learning algorithm [134]. The system was evaluated in 90 GDM patients. The results showed a reduction in the time devoted by clinicians to patients and in face-to-face visits per patient.

Six other studies have proposed alternatives to the manual creation of patient care workflows. The studies offer support for the design and deployment of diabetes management protocols, as well as ways to continuously improve patient tracking throughout the entire process. Cleveringa et al presented a system aimed at decreasing cardiovascular risk of T2D patients by optimizing patient care workflows [135,136]. The authors validated their system by administering questionnaires to 3391 T2D patients. Miller et al used a machine learning approach to extract information from drug prescriptions from electronic health record (EHR) data and identify factors associated with patient care flow deviations [23]. Another DSS with care flow tools was presented in the work of Alotaibi et al [137]. This system focuses on the management of T2D patients using advanced features, such as computerized alerts and reminders. It was tested in 20 T2D patients for 6 months and resulted in reduced HbA_1c_ levels and improved diabetes awareness. Fernandez-Llatas et al proposed using data mining methods to enable the dynamic design of care protocols but highlighted the need for mechanisms to reduce the Spaghetti Effect and make DSSs usable by experts [138]. Contreras et al developed a diabetes management system to integrate a series of AI models and tools with an engine to manage diabetes patient care flows [139]. Finally, Suh et al proposed a dynamic care flow system that applied data clustering together with rule mining techniques to prioritize required user tasks [140].

Other tools have been proposed for improving daily-life support for diabetes therapies. Four different tools have been designed to analyze online discussion forums and social networks to extract relevant information. First, Grieves et al compared multiple machine learning techniques (decision trees, SVM, bagging, and Bayes) to analyze patients’ online comments with the aim of predicting patients’ assessment of hospital performance [141]. Second, Valdez et al proposed using a k-means clustering analysis to identify communication patterns both on and off Facebook [142]. They validated their tool in a cohort of 700 T2D patients. Third, Chen et al proposed clustering based on repeated bisecting k-means with the goal of obtaining patient experience information, including emotional and temporal aspects of diabetes management [143]. Finally, Hamon et al proposed using NLP methods to extract information about patients’ skill in managing diabetes [144].

Furthermore, tools have been developed to analyze clinical appointments, medication, and therapy adherence. For example, a machine learning approach to examine medication adherence thresholds and risk of hospitalization was implemented [145]. The system implemented in the study reported by Fioravanti et al promotes patient empowerment and adherence to therapy based on the automatic generation of feedback messages [146]. Greaves et al proposed a clinical DSS that issues medication interaction alerts based on clusters with similar management recommendations [147]. A fuzzy approach was also presented by Eghbali-Zarch et al to address the problem of medication selection in T2D patients [148]. Finally, Kurasawa et al proposed a machine learning algorithm to predict missed clinical appointments and help patients continue regular doctor visits [149].

## Discussion

By systematically examining high-quality articles in the PubMed database, we identified a series of studies with the goal of evaluating the latest efforts of AI-enabled solutions for diabetes management. The topics we reviewed suggest that prediction and prevention are currently being revitalized and reinforced by AI applications, whereas “safety and failure detection” has been less extensively reviewed, constituting fewer than 6% of the studies we encountered. Similarly, few investigations have delved into the application of AI techniques to early detection of critical issues such as exercise, meals, infusion set failures, and so forth. Exploiting the latest AI techniques to improve the safety of both AP systems and open-loop tools has the potential to dramatically improve performance. By contrast, research on closed-loop systems, representing 31 out of 141 of the reviewed studies (22%), has been the most productive area for AI applications. Most of these efforts addressed fuzzy techniques, but the application of other methodologies has begun to attract increasing interest. In our opinion, researchers in this field should continue to take advantage of the latest improvements in AI and to combine them with development of the AP. A considerable number of the reviewed studies, 41 out of 141, investigated BG, either to develop models that enable accurate predictions of BG concentrations (27 studies) or to detect possible BG events (14 studies). Multiple studies reported accurate prediction and detection tools that promise to improve management resources for current and future therapies. These tools include bolus advisors, as well as both lifestyle and patient stratification.

Our findings show the increasing importance of AI methods for diabetes management. We think these methods will encourage further research into the use of AI methods to extract knowledge from diabetic data. In general, the most striking advances in the application of AI techniques come from data-driven methods that learn from large datasets. The ability to collect information from individual diabetic patients has led to a shift in diabetes management systems; accordingly, systems that lack access to valuable data will face substantial hurdles. Diabetes management is geared toward tailored management of therapies, at the level of smaller strata of patients or even individuals. Thus, management protocols provided to diabetic patients should be tailored to address their needs at various points during their illness. Furthermore, the availability of genetic data, such as that provided by metabolomics analysis, has also empowered the application of AI methods to personalization of diabetes management.

The increased availability of digitized health data from diabetic populations, along with the emerging applications of AI and research trends such as the AP and personalized medicine, suggests that we are moving toward a new paradigm for management of diabetes. This new outlook proposes to achieve custom delivery of diabetes care while tailoring professional practices, medical decisions, and treatments to individual patients. On the other hand, the inclusion of intelligent algorithms in decision making has ethical implications that should be addressed by physicians and scientists. The ethical risks associated with the release of personal data should also be investigated. For example, the increasingly frequent use of health apps and the potential use of tools based on AI by insurance companies could lead to discrimination or the exclusion (or both) of some citizens from health services.

A large number of studies have already been published on the application of AI to diabetes in a broad range of management domains. Our dive into PubMed demonstrates an acceleration in the pace of research on AI-powered tools designed to predict and prevent the complications associated with diabetes. Although the available technologies and methods for diabetes management are growing exponentially in terms of quantity and quality, the potential of AI to boost effective and accurate management of diabetes has already been demonstrated in both open- and closed-loop therapies. Research in this field should continue and should seek to discover the opportunities and advantages of applying AI methodologies in diabetes management that differentiate these strategies from other classical approaches.

## Abbreviations

AI: artificial intelligence
ANN: artificial neural network
AP: artificial pancreas
BG: blood glucose
CGM: continuous glucose monitoring
CSII: continuous subcutaneous insulin infusion
DSS: decision support system
DT: decision tree
DL: deep learning
EA: evolutionary algorithm
FL: fuzzy logic
GA: genetic algorithm
GDM: gestational diabetes
KDD: knowledge discovery in databases
KNN: k-nearest neighbor
MDI: multiple daily injection
NB: Naïve Bayes
NLP: natural language processing
PH: prediction horizon
RA: regression algorithm
RF: random forest
RMSE: relative mean square error
SVM: support vector machine
T1D: type 1 diabetes
T2D: type 2 diabetes
